# Failure Characteristics and Mechanism of Nano-Modified Oil-Impregnated Paper Subjected to Repeated Impulse Voltage

**DOI:** 10.3390/nano8070504

**Published:** 2018-07-07

**Authors:** Potao Sun, Wenxia Sima, Dingfei Zhang, Xiongwei Jiang, Huangjing Zhang, Ze Yin

**Affiliations:** 1 State Key Laboratory of Power Transmission Equipment & System Security and New Technology, Chongqing University, Chongqing 400030, China; cqsmwx@cqu.edu.cn (W.S.); jiangxiongweicqu@163.com (X.J.); Huangjing_Zhang@cqu.edu.cn (H.Z.); cquyinze@cqu.edu.cn (Z.Y.); 2 College of Engineering Materials, Chongqing University, Chongqing 400030, China; zhangdingfei@cqu.edu.cn; 3 Department of Electrical and Computer Engineering, University of Florida, Gainesville, FL 32611, USA

**Keywords:** nano-oil-impregnated paper (NOIP), space charge, accumulative effect, atomic force microscope, micropore structure

## Abstract

Nano-modification is a prospective method for improving the electrical properties of transformer oil. In most situations, transformer oil combined with cellulose paper is used to construct an insulation system for power equipment, such as power transformers. However, the influence of nanoparticles on the electrical performance of oil-impregnated paper is still unclear. Therefore, in this paper, we identify the failure characteristics of both fresh and nano-modified oil/paper. Specifically, the accumulative failure characteristics of nano-oil-impregnated paper (NOIP) are experimentally determined. The space charge distribution and trap characteristics of fresh paper and NOIP were measured, and the effect of nanoparticles on the space charge behavior are then analyzed. Finally, we measure the microstructure of fresh paper and NOIP subjected to repeated impulses. The test results indicate that nano-titanium oxide (TiO_2_) particles have a limited effect on the breakdown voltage of NOIP. However, the particles can dramatically improve the resistant ability of NOIP against repeated impulses. For the NOIP with a nano-concentration of 0.25 g/L, the improvement reaches 62.5% compared with fresh paper. Under repeated applications of impulse voltages, the space charge density of NOIP is much lower than that of fresh paper. The deep trap density of NOIP is much higher than that of fresh OIP, whereas shallow trap density is relatively lower. Micropores are generated in paper insulation subjected to repeated impulses. The amount of the generated micropores in NOIP is lower than that in fresh paper. Nano-TiO_2_ particles suppress the accumulation of space charge in the oil paper insulation, which weakens the electric field distortion in the dielectric. However, nanoparticles reduce the accumulative damage caused by repeated impulses. The above two points are considered the main reasons to improve the resistant ability against repeated impulses.

## 1. Introduction

A particularly novel approach to improving the properties of transformer oil has been developed by suspending nanoparticles in transformer oil. The test results indicate that the insulating performance, heat dissipation performance, and anti-aging performance of nano-modified oil are improved to different degrees [1,2,3,4,5,6]. Segal et al. successively found that the breakdown voltages of nano-modified transformer oils increase by different degrees (the test nanoparticles include Al_2_O_3_, TiO_2_, Fe_3_O_4_, SiO_2_, carbon nanotube and so on) [7,8,9,10,11]. The breakdown voltage of nano-oils can even reach 3.3 times that of pure oil [12]. Some disadvantages of nano-oil were also found including increasing dielectric loss [13], agglomeration [14], decreasing the negative breakdown voltage [15] and migration under external electric/magnetic field [16]. Although the industrial application of nano-fluids still requires development due to these limitations, the application of nanoparticles in the insulation area is still considered as having potential [17,18,19,20,21].

For over 100 years, cellulose paper combined with insulation oil have been used as the insulation system for power transformers. Cellulose paper is impregnated with transformer oil to create a composite insulation called oil-impregnated paper (OIP) with good electrical performance. Many studies have focused on nano-modified transformer oil, but cellulose paper impregnated with nano-modified transformer oil (nano-oil-impregnated paper, NOIP) has rarely been studied, including the effects of nano-modified transformer oil on the performance of NOIP and the mechanism of nanoparticles.

Among the characteristics of OIP, breakdown under impulse voltage is an important indicator for evaluating the modification effect of nanoparticles. We have been monitoring a 110 kV power transformer in China for a long time. Monitoring results revealed that more than 50 lightning impulses invaded this transformer each year [22,23,24,25]. The number of recorded switching impulses was even higher at four to five per day. If we assume the lifetime of a power transformer is 30 years, this transformer will be subjected to more than 40,000 impulses. Such repeated impulses may cause irreversible damage to insulation dielectrics, or even premature failure. This phenomenon is known as accumulative dielectric failure, which was studied in our previous work [21,22,23,24,25,26]. Therefore, focusing on the above problems, the breakdown characteristics and accumulative failure characteristics of both OIP and NOIP were determined in this work. The corresponding mechanisms behind the observed phenomena were also analyzed.

Here, we present our work in this area with respect to the effect of nano-TiO_2_ particles on the accumulative failure of OIP. First, we determine the breakdown characteristics of nano-modified transformer oil (NMTO), fresh transformer oil (FTO), NOIP, and OIP. The accumulative failure characteristics of NOIP and OIP are also identified. Then, we measure the space charge distribution in OIP and NOIP subjected to repeated impulses. The total space charge amount and space charge decay rate of both OIP and NOIP with different nanoparticle concentrations are calculated. Finally, we measure the surface morphology and micropore structure of fresh OIP and OIP/NOIP subjected to repeated impulses.

The remainder of the paper is structured as follows. The experimental setup is presented in Section 2, and the tests for the breakdown characteristics are described in Section 3. The mechanisms are discussed in Section 4, and finally conclusions end the paper in Section 5.

## 2. Experimental Setup

### 2.1. Preparation of Nanofluids

Karamay transformer mineral oil (#25) was selected as the base oil. All the test oil was filtered by a vacuum oil-filter to remove the impurities. The technical specifications of the test materials satisfy the international standard ASTM D3487-2000(II) [27] and the value recommended by the CIGRE working group 12.17 [28]. The nanofluid was prepared as follows.

Stearic acid was used as surface modifier. TiO_2_ nanoparticles were mixed with stearic acid and placed in a water bath case at 60 °C. The mixture was stirred by a mechanical agitator for 2 h. When the mixture was evenly mixed, we added transformer oil to the beaker containing the nano-TiO_2_ mixture. Ultrasonic dispersion was then conducted for 45 min. All the nano-modified transformer oil (NMTO) was dried at 90 °C for 48 h in a vacuum drying oven. After the pre-treatments, all the samples were sealed in glassware stored in a cool, dry, isolated, and well-ventilated storage area.

**Figure 1:**
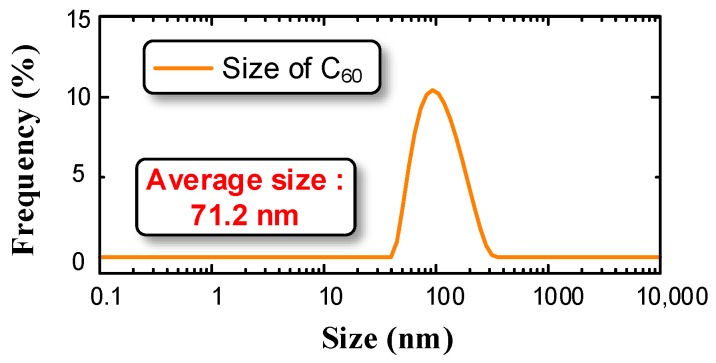
Size distribution of nano-titanium oxide (TiO_2_) particles.

We measured the size distribution of nano-TiO_2_ particles contained in nanofluid as shown in Figure 1. The average particle size was 71.2 nm.

A laser beam was shot into the nanofluid to measure the stability of nanoparticles. Light-transmission coefficient of nanofluids, which is the ratio of output light intensity to incident light intensity, was measured every 8 h. The light-transmission coefficient remained stable after 120 h. The test results indicate that the nano-TiO_2_ particles dispersed steadily in the base oil.

The pre-treatments of OIP and NOIP were as follows: first, cellulose paper and transformer oil/NMTO were separately dried at 90 °C for 48 h in a vacuum drying oven. Oil impregnation was then conducted for another 48 h using FTO/NMTO that had been purified and vacuum degassed.

### 2.2. Breakdown Test Platform and Test Method

**Figure 2:**
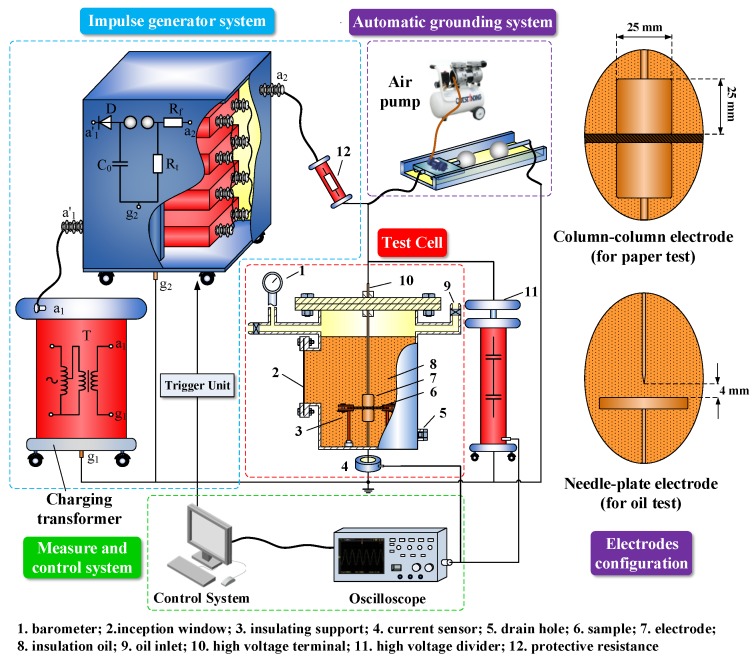
Sketch of impulse voltage test setup.

The test setup is shown in Figure 2. A cylindrical steel test cell with a good sealing performance was used to hold the electrodes and test samples. Needle-plate electrodes and column-column electrodes were selected for the oil breakdown test and OIP breakdown test, respectively [29,30,31]. Two copper rods connecting to the electrodes were fixed on the test cell, serving as the high voltage terminal and grounding terminal. Given that oil paper is normally used as multilayer insulation in power transformers, three layers of the OIP samples (thickness: 55 μm per layer) were placed between the electrodes. An impulse generator was used to output standard impulse voltage (30 kJ/400 kV). Voltage and current signals were measured by a high voltage divider and current sensor, respectively. The data were recorded by a TDS2024C impulse analysis system. The parameters of the electrodes are shown in Table 1.

The up and down method was used to determine the 50% breakdown voltage (U_50_) of the test samples [32,33]. The number of valid data for each test was more than 50. The test procedure for the accumulative effect test is outlined in detail in our previous work [22,23,24,26].

### 2.3. Measurement Parameters

#### 2.3.1. Pulsed Electro-Acoustic (PEA)

**Figure 3:**
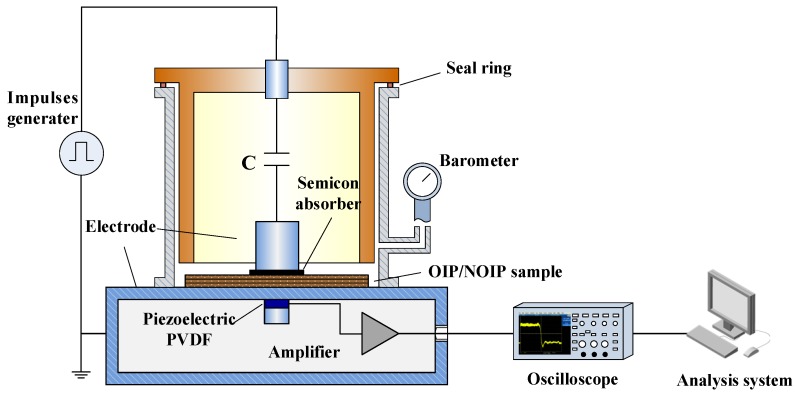
Schematic diagram of Pulsed Electro-Acoustic (PEA) test system.

The space charge distribution of NOIP/OIP subjected to repeated impulse voltage was measured using the PEA method. The schematic diagram of the PEA test system is shown in Figure 3. Our testing procedures are as follows:

1. Apply repeated impulse voltage to the OIP sample and NOIP sample with different nanoparticle concentrations.
2. Transfer the test sample to the PEA measurement system. The transfer interval time is less than 15 s.
3. Measure the space charge distribution of NOIP/OIP sample at a specified time.

#### 2.3.2. Thermally Stimulated Current (TSC)

**Figure 4:**
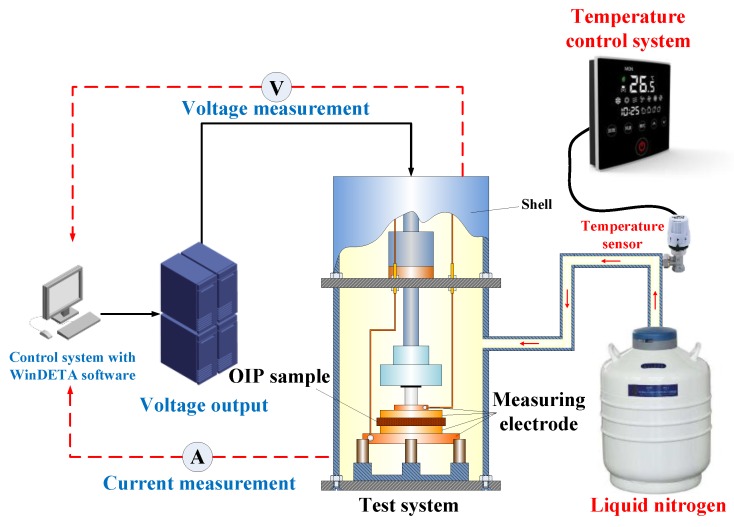
Thermally Stimulated Current (TSC) measurement system.

Thermally Stimulated Current (TSC) was measured by a Novocontrol TSC measurement system that is composed of microcurrent measurement system, DC electrical source, temperature control system, and a temperature/current real-time recording system. Liquid nitrogen was used to control the temperature. The structure of the TSC measurement system is shown in Figure 4.

#### 2.3.3. Atomic Force Microscope Measurement (AFM)

The maximal precision of the atomic force microscope (IPC-208B, Oxford Instruments company, Concord, MA, USA) used in the test is 0.1 nm in cross direction and 0.01 nm in lengthwise direction, and the maximal scanned area is 90 μm × 90 μm.

#### 2.3.4. Mercury Intrusion Micropore Structure Measurement

The micropore structures of OIP and NOIP were determined using the mercury intrusion method. The measuring principle was as follows. Mercury can easily fill the micropores inside a solid under applied pressure. The energy consumed by the increase in mercury equals to the work done by external forces, which is the surface free energy of the mercury-solid interface under the same thermodynamic condition. The external pressure equals the capillary pressure: the smaller the pore, the higher the capillary pressure, and thus the higher the corresponding external pressure. By measuring the pressure and volume of the mercury, we were able to calculate the diameter distribution, pore surface areas, and porosity.

A fully automatic mercury injection apparatus was used in this paper. The maximum pressure was 414 MPa and the measurement range of the pore radius varied from 6 nm to 339 μm.

## 3. Effect of Nanoparticles on the Electrical Performance of NMTO and NOIP

### 3.1. Breakdown Characteristics of NMTO

**Figure 5:**
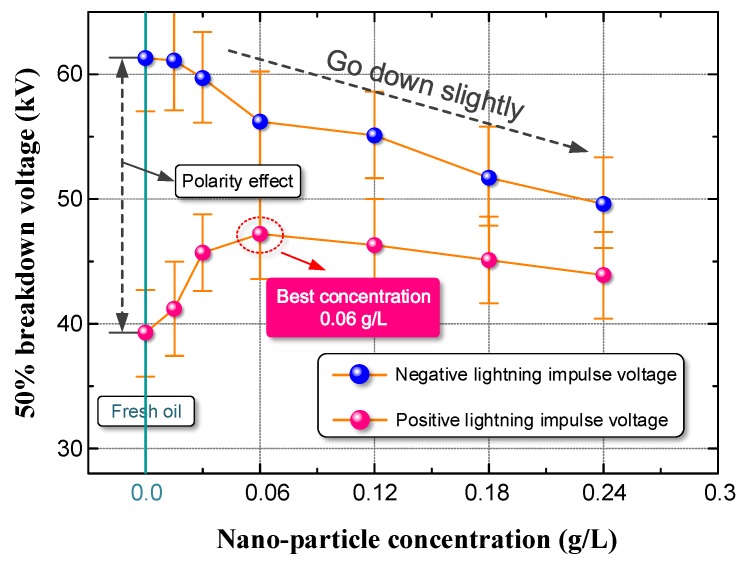
The 50% breakdown voltage (U_50_) of fresh transformer oil (FTO) and nano-modified transformer oil (NMTO) under standard lightning impulse voltage.

Nanoparticles are thought to have a positive effect on the liquid dielectrics’ electrical performance. In this section, we first determine the U_50_ of FTO and NMTO with different nanoparticle concentrations. The test voltage was the standard lightning impulse voltage (1.2/50 μs). The test results are shown in Figure 5.

Due to the polarity effect, the U_50_ for the negative voltage was much higher than for the positive voltage. Please note that as the nanoparticle concentration increases, the U_50_ increases first and then decreases slightly when the positive impulse voltage is applied to the NMTO. The U_50_ of NMTO reached a maximum when the concentration of nano-TiO_2_ particles was 0.06 g/L. However, when the negative impulse voltage was applied to the NMTO, the U_50_ of NMTO decreased gradually as the nanoparticle concentration increased.

### 3.2. Breakdown Characteristics of NOIP

**Figure 6:**
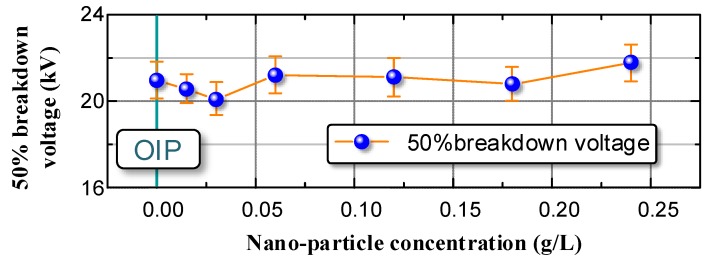
U_50_ of oil-impregnated paper (OIP) and nano-OIP (NOIP) with different nano-TiO_2_ particle concentrations.

According to the pre-treatment procedure we obtained NOIP samples with different nano-TiO_2_ particle concentration and determined their U_50_. The test results are summarized in Figure 6.

Notably, although nano-TiO_2_ particles exert a significantly positive effect on the transformer oil’s electrical performance, its influence on the cellulose paper was limited. The U_50_ of NOIP barely changed when the nano-TiO_2_ particles concentration varied. This phenomenon may depend on the breakdown mechanism of cellulose paper: the OIP has many pores. Since the relative permittivity of oil is much lower than that of cellulose paper, the electric field intensity in the oil pores is extremely high when lightning impulse voltage is applied to the OIP. Breakdown occurs first in these oil pores. When the breakdown channel runs through the oil pores, the impressed voltage is completely applied to the cellulose dielectric. If the impressed voltage exceeds the critical value, the OIP breaks down; otherwise, the arc in the oil pores extinguishes. Therefore, the breakdown characteristics of OIP depend on the electrical performance of cellulose rather than the transformer oil. Although the nano-TiO_2_ particles enhanced the electrical performance of the oil pores, the breakdown voltage of the cellulose fibers remains unchanged. Hence, the U_50_ of NOIP barely changed when the nano-TiO_2_ particles concentration varied.

### 3.3. Accumulative Failure Characteristics of NOIP

**Figure 7:**
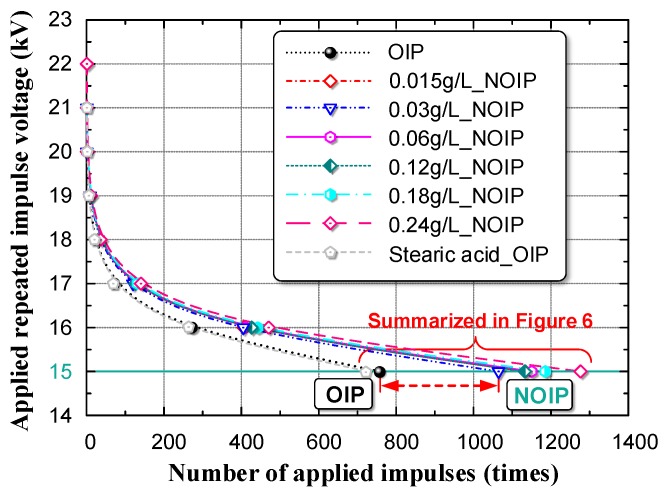
Amplitude of applied impulse voltage (V) and number of applied impulses before breakdown (N), the V–N characteristics, of OIP and NOIP with different nano-TiO_2_ concentrations.

The relation between amplitude of applied impulse voltage (V) and number of applied impulses before breakdown (N), the V–N characteristic, is an essential index reflecting the electrical performance of OIP [22,23,24]. We identified the V–N characteristics of OIP and NOIP with different nano-TiO_2_ particle concentrations. The test results are shown in Figure 7.

**Figure 8:**
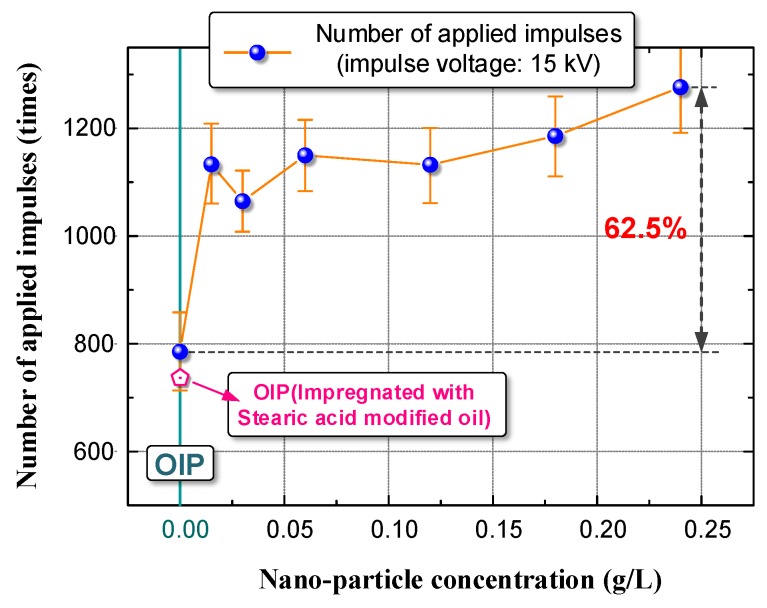
Number of applied impulses before breakdown occur for OIP and NOIP.

Notably, the V–N characteristics curve of OIP differs from that of NOIP. When the amplitude of applied voltage was relatively low, the difference among the V–N characteristics curves became obvious. This observed phenomenon indicates that nanoparticles play a positive role in the accumulative failure process of OIP and improve the OIP’s resistant ability against repeated impulses. To exclude the effect of the surface modifier (stearic acid) on the test results, we determined the V–N characteristics of stearic-acid-oil mixture-impregnated paper, represented by the grey dashed line in Figure 7. The grey dashed line coincides with the black dotted line, which indicates that the surface modifier has no effect on the V–N characteristics. Based on the test results presented in Figure 7, we summarized the number of applied impulses before breakdown occurred (N) in Figure 8. The amplitude of the applied voltage was 15 kV.

Figure 8 shows that the *N* of NOIP significantly improved compared with that of OIP. As the nanoparticle concentration increased, the *N* of NOIP decreased first, and then increased slightly. The *N* was 62.5% higher for NOIP (0.25 g/L nanoparticle concentration) compared to that of OIP. In the following sections, we conduct further studies to reveal the affecting mechanism of nanoparticles on the resistant ability of NOIP against repeated impulses.

## 4. Analysis and Discussion

### 4.1. Failure Mechanism of OIP under Repeated Impulses

Many studies have proven that nanoparticles have a positive effect on the electrical performance of liquid dielectrics [1,2,34,35,36]. Many scholars think that surface charges are formed on the nanoparticles via induction or polarization. This leads to the forming of a potential barrier surrounding the nanoparticles. Therefore, nanoparticles can easily capture electrons in the steamer, thereby reducing the number of electrons in the steamer. This reduces the speed of the streamer and improves the breakdown voltage of the liquid dielectric. The modification mechanism of nanoparticles on the transformer oil are being widely studied. We mainly focused on the modification mechanism of nanoparticles on oil paper insulation in this paper.

To reveal the mechanism by which nano-TiO_2_ particles influence the accumulative failure characteristics of cellulose paper, we first wanted to determine the accumulative failure mechanism of OIP. In our previous study, space charge accumulation and accumulative damage caused by repeated lightning impulses were considered two main reasons for accumulative failure of OIP [26]:

1. Space charge accumulation: under repeated lightning impulse voltage, positive space charges are injected into the OIP sample and migrate toward cathode. In this process, some of the positive space charges are trapped in the dielectric. Conversely, negative charges, which mainly consist of electrons, can easily pass through the dielectric and be neutralized in the anode. The existence of positive charges in the bulk strengthen the electric field near the cathode, thereby contributing to the breakdown of the OIP.
2. Dielectric accumulative damage: when the impulse voltage is applied to the OIP sample, the electric field intensity in the oil pores becomes extremely high. Partial discharge occurs repeatedly in the oil pores. When the charged particles and electrons collide, some of the cellulose fibers break and form a new substance. In this process, the cellulose paper suffers unrecoverable damage, thereby leading to the decrease in the breakdown voltage of the OIP [22].

Based on the above research results, we attempted to reveal the influencing mechanism of nano-TiO_2_ particles on cellulose paper by studying the space charge distribution and dielectric accumulative damage characteristics of NOIP.

### 4.2. Space Charge Distribution in OIP and NOIP

**Figure 9:**
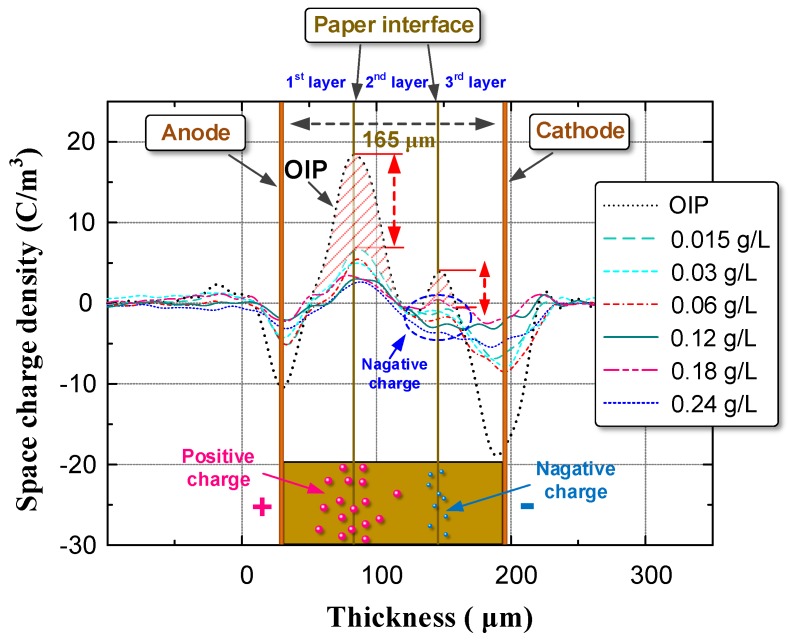
Space charge distribution in OIP and NOIP.

According to the test procedure in Section 2.3, we identified the space charge distribution in OIP and NOIP. The amplitude and number of applied impulses were set to 15 kV and 500, respectively. The test results are shown in Figure 9.

Notably, in OIP, large quantities of charges were injected into the bulk from the repeated impulses. Positive charges migrated toward the cathode and accumulated on the paper interface between the first and second layers. Some of the positive charges break through the interface barrier and accumulate on the paper interface between the second and third layers. Compared with OIP, the space charge distribution in NOIP was different in two aspects:

1. The space charge density of NOIP is much lower than that of OIP.
2. Positive charges were detected on the paper interface between the 2nd layer and the 3rd layer in the OIP sample. Whereas in the NOIP sample, negative charges were observed on this paper interface.

Total charges in the dielectric, independent of polarity, can be calculated by the following equation [37].

(1) $$Q\left(t\right)=\int_{0}^{d}\left|\rho \left(x,t\right)\right|Sdx$$

where *ρ* is the charge density, *x* is the position, *d* is the thickness of the sample, and *S* is the effective area of the specimen. 

**Figure 10:**
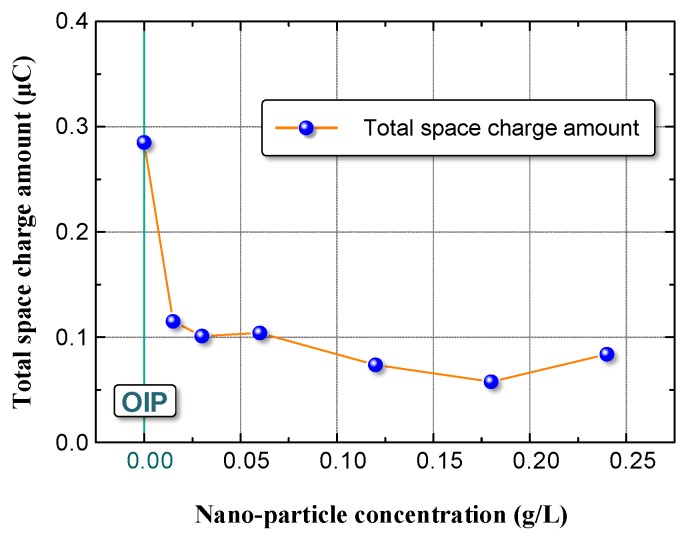
Total charge amount in the OIP sample and NOIP sample.

Based on the test results presented in Figure 9, we calculated the total charges in the OIP and NOIP samples as shown in Figure 10.

The total space charge amount in NOIP was considerably lower than in OIP. As the nanoparticle concentration increased, the total space charge amount in NOIP decreased first, and then increased slightly when the nanoparticle concentration exceeded 0.18 g/L.

**Figure 11:**
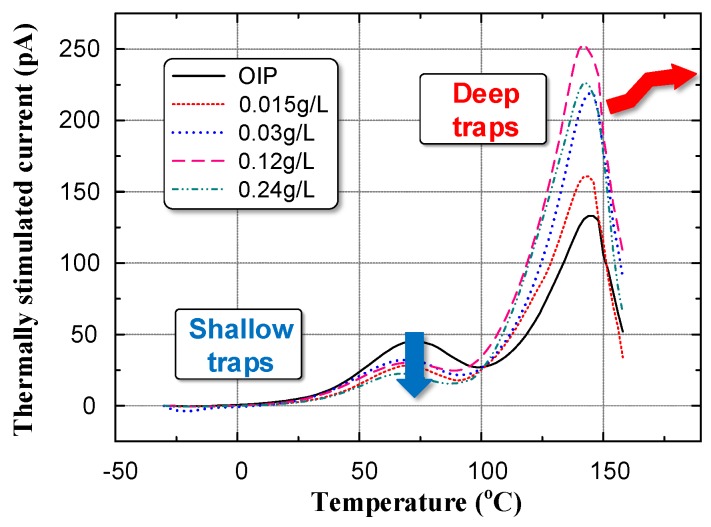
Thermally stimulated current of OIP and NOIP.

We measured the TSC of both OIP and NOIP to determine the trap characteristics of the dielectrics. The test results are shown in Figure 11.

We chose Gaussian fitting to separate the current peaks. Then the average energy level of shallow traps and deep traps were calculated using [38,39]:

(2) $$E_{tr}=\frac{2.47T_{m}{}^{2}k_{B}}{\Delta T}$$

**Figure 12:**
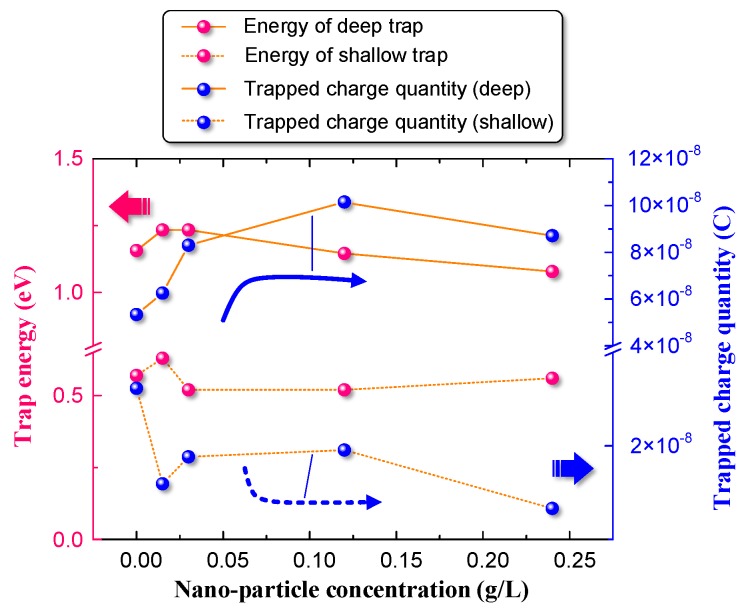
Effects of nanoparticle concentration of the trap parameters.

The quantity of trapped charge was calculated using the following equation [38,39]:

(3) $$Q_{tr}=\int_{t_{0}}^{t_{1}}I(t)dt=\frac{60}{\beta }\int_{T_{0}}^{T_{1}}I(T)dT$$

where *β* is the temperature rate in K/min, *T_m_* is the temperature corresponding to the crest in K, and Δ*T* is the temperature difference corresponding to the half-values. The trap energy and quantity of trapped charge are summarized in Figure 12.

As shown in Figure 12, the nanoparticles had limited effects on the energy levels of traps. The deep trap energy level and shallow trap energy level fluctuated near 0.57 eV and 1.16 eV, respectively. The amount of trapped charges, related to trap density in the dielectric, changed as the nanoparticle concentration varied. As the nanoparticle concentration increased, the deep trap density increased initially and then decreased slightly. However, the shallow trap density decreased with increasing nanoparticle concentration. Pourrahimi et al. proved that a “strong” interface forms between the dielectric and nanoparticle [40]. The interfaces introduced by nanoparticles are directly related to the trap characteristics of the nano-composite dielectric. Moreover, polar groups on the particle surface and a disturbed polymer molecular order at the particle surface were also considered to provide charge traps [40]. Therefore, the change in trap density in the dielectric is considered to be dominated by the nanoparticles, which change the charge transportation characteristics. Based on the work by Nelson et al., two possible nanoparticle mechanisms that could affect charge transportation are presented as follows [41,42].

Firstly, when two different materials contact each other, free carriers flow from one material to the other due to their different Fermi levels. This free carrier transportation ends when a new balance is established. Charges are hard to inject from a metal electrode into a dielectric due to the barrier potential between these two materials. When the external electric field exceeds a critical value, Schottky charges are injected into the dielectric. The electric field between column-column electrodes is quasi-homogeneous, which is much lower than the critical value of field-emission tunneling. The injection current is expressed as [43,44]:

(4) $$I=i_{0}e^{-\Phi /kT}\times e^{\frac{e\sqrt{eE_{e}/4\pi \varepsilon_{0}\varepsilon_{r}}}{kT}}$$

where *Φ*(*x*) is the barrier potential for charge injection, *E_e_* is the electrode field, and *ε_r_* is relative permittivity of the samples. The barrier potential at the electrode-dielectric interface is expressed by the following equation [43,44]:

(5) $$\Phi (x)=\Phi_{m}-\chi -\frac{q^{2}}{16\pi \varepsilon_{0}\varepsilon_{r}x}-qE_{e}x$$

where *Φ_m_* is the work function of the metal electrode, *χ* is the electron affinity, *q* is the electron charge, and *x* is the distance.

Free charges are captured by the traps. The trapping time, which is the time between trapping and de-trapping, increases with increasing trap depth [45]. As shown in Figure 12, nanoparticles introduce deep traps into the dielectric. More deep traps emerge near the electrode, which capture homocharges and thereby weaken the local electric field near the electrode [42,46]. According to Equation (5), such a decrease in the electrode field contributes to increasing the injection barrier potential, which leads to a decrease in the space charge density in NOIP.

**Figure 13:**
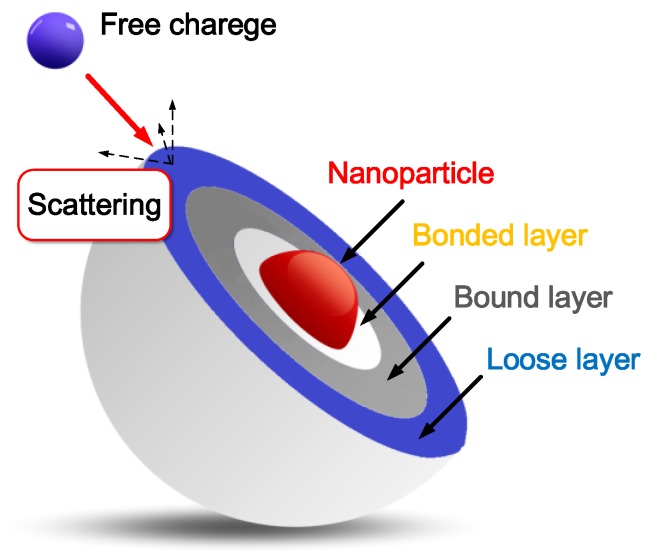
Multicore model and scattering of nanoparticles.

Secondly, Tanaka et al. proposed a multilayer description of the particle-polymer interface, as shown in Figure 13. This interface consists of a “bonded” layer closest to the particle, a “bound” layer further out, and finally a “loose” layer [47]. The interfacial regions are claimed to be scattering centers for charge carriers. Free charges migrate in the dielectric, motivated by the external electric field. Some of the free charges are scattered at surfaces of the nanoparticles, which reduces the energy of free charges and results in an overall reduced charge accumulation (Figure 13) [42,48]. 

**Figure 14:**
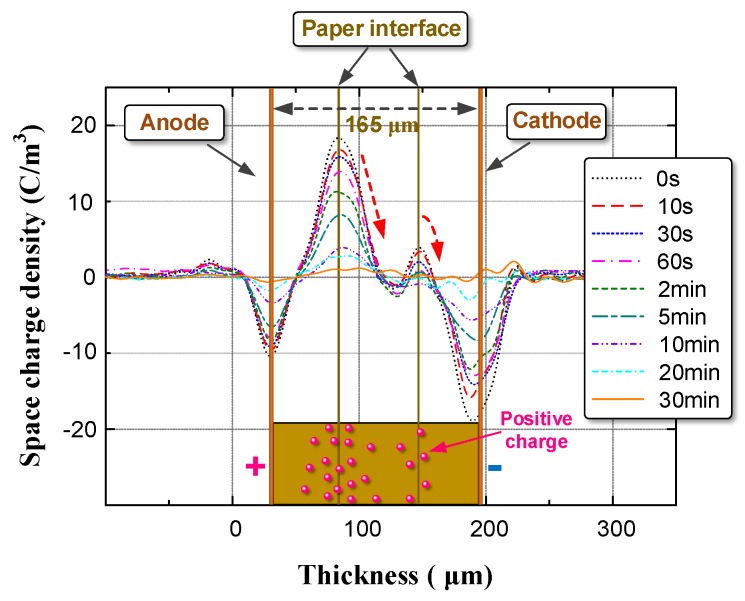
Space charge decay characteristics of OIP.

**Figure 15:**
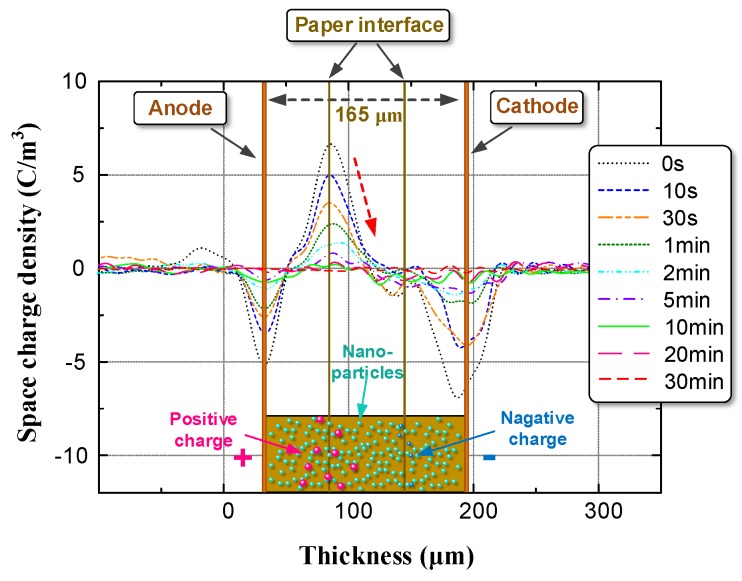
Space charge decay characteristics of NOIP at a nanoparticle concentration of 0.015 g/L.

We then determined the space charge decay characteristics of OIP and NOIP with a nanoparticle concentration of 0.015 g/L, as shown in Figure 14 and Figure 15.

**Figure 16:**
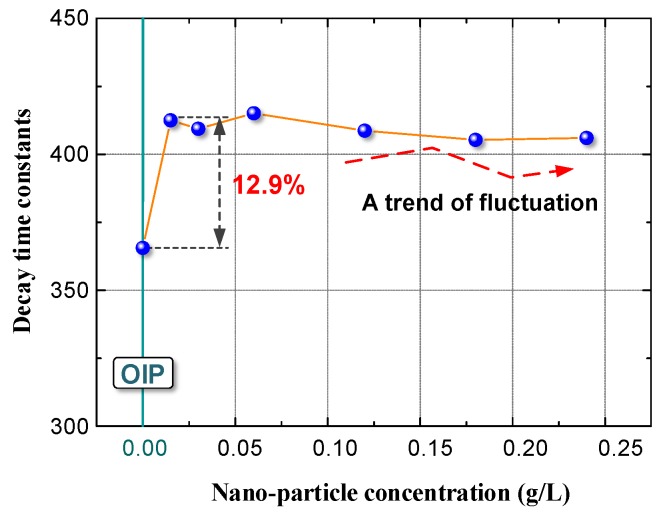
Decay time constants of the OIP and NOIP samples.

Please note that the space charges in the dielectric decayed rapidly once the sample was short circuited. An exponential function was used to characterize the relation between total charges and decay time.

(6) $$Q(t)=Q_{0}+Ae^{\frac{-\tau }{t}}$$

where *t* is decay time and *τ* is the decay time constant. The decay time constants of space charges in OIP and NOIP (0.015 g/L) were 365.3 and 412.5, respectively. Therefore, the space charge decay rate for OIP was 12.9% higher than that of NOIP (0.015 g/L). To reveal the effects of nanoparticles on the space charges dissipation, space charge decay characteristics of NOIP with various nanoparticle concentrations were determined. The decay time constants of the NOIP samples are presented in Figure 16.

Please note that the decay constants of the NOIP samples are higher than that of the OIP sample. However, nanoparticle concentration demonstrated a limited effect on the space charge dissipation process. The trend in the decay constants of the NOIP fluctuated with increasing nanoparticle concentration. Deep traps in the dielectric are thought to reduce the mobility of free charges and increase the charge decay rate by capturing free charges. Therefore, the decay constants of the NOIP are higher due to high deep trap density. However, we still cannot present a rational explanation for the limited effects of nanoparticle concentration on the decay constant.

We conclude from the above test results that the nanoparticles enter cellulose paper during the impregnation process. Due to interfaces introduced by the nanoparticles, a considerable number of deep traps emerge in the NOIP dielectric that capture free charges near the electrode. This behavior weakens the local electric field near the electrode and thereby suppresses the injection of space charges. Moreover, a scattering effect caused by nanoparticles can reduce the energy of free charges and thereby suppress the charge accumulation in the dielectric. According to our previous study, positive charges in the bulk can strengthen the electric field intensity near the cathode, which contributes to the accumulative failure of the OIP sample. Therefore, we suspect that by reducing the space charge density in cellulose paper, nanoparticles improve the OIP’s resistant ability against repeated impulses, which led to the results presented in Figure 7. In addition, in NOIP, negative charges were detected near the cathode, which can further reduce the electric field near the cathode and improve the OIP’s resistant ability against repeated impulses.

### 4.3. Microstructure of OIP/NOIP

To study the effect of repeated impulses on the microstructure of OIP and NOIP, we measured the surface micromorphology and micropore structure of OIP/NOIP subjected to repeated impulses.

#### 4.3.1. Surface Micromorphology

**Figure 17:**
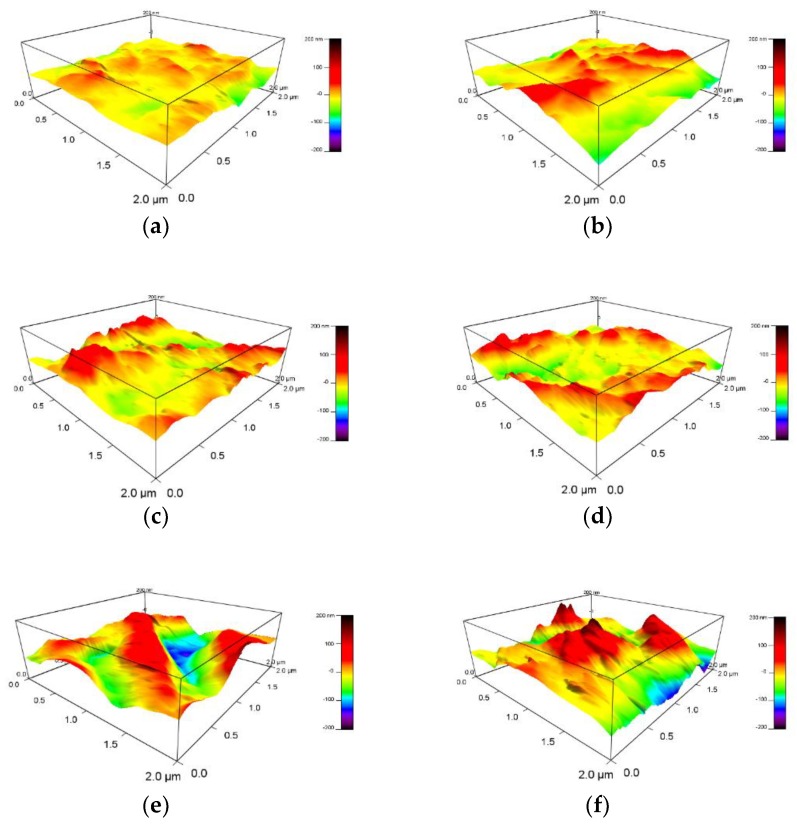
Surface morphology of OIP and NOIP: (**a**) fresh OIP, (**b**) fresh OIP subjected to 500 impulses, (**c**–**f**) NOIP subjected to 500 impulses, with nanoparticle concentrations of 0.015, 0.06, 0.12, and 0.24 g/L, respectively.

We obtained the surface micromorphology of fresh OIP and OIP/NOIP subjected to repeated impulses by using an atomic force measurement (AFM) system. The scanned area was 2 μm × 2 μm. The amplitude and number of applied impulses were set as 15 kV and 500, respectively. The test results are shown in Figure 17.

Notably, the surface of fresh OIP is flat and smooth. When we applied repeated impulses to the OIP sample, some red bumps arose on the surface of the dielectric, as shown in Figure 17b. Compared with that of OIP, the surface of the NOIP subjected to repeated impulses was much rougher. Some relatively high peaks were observed on the surface of NOIP samples whose nanoparticle concentrations were 0.015 and 0.06 g/L. When the nanoparticle concentration increased further to 0.12 and 0.24 g/L, the red peaks became higher and some pits appeared on the surface of the dielectric.

**Figure 18:**
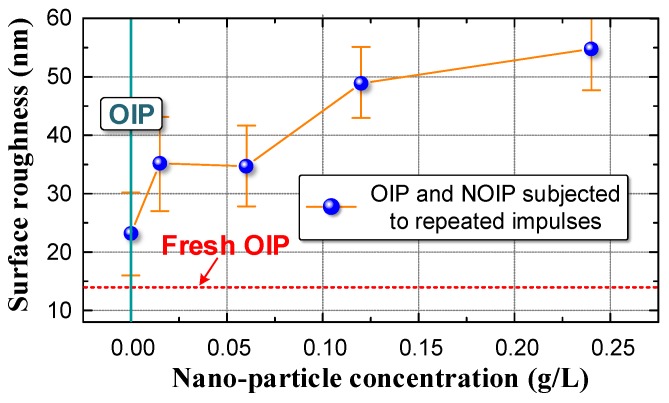
Relation between surface roughness and nanoparticle concentration.

To quantitatively analyze the change of micromorphology, we randomly measured eight points on a test sample and calculated the average values of surface roughness (RMS) [49,50,51]. The relationship between surface roughness and nanoparticle concentration is plotted in Figure 18.

**Figure 19:**
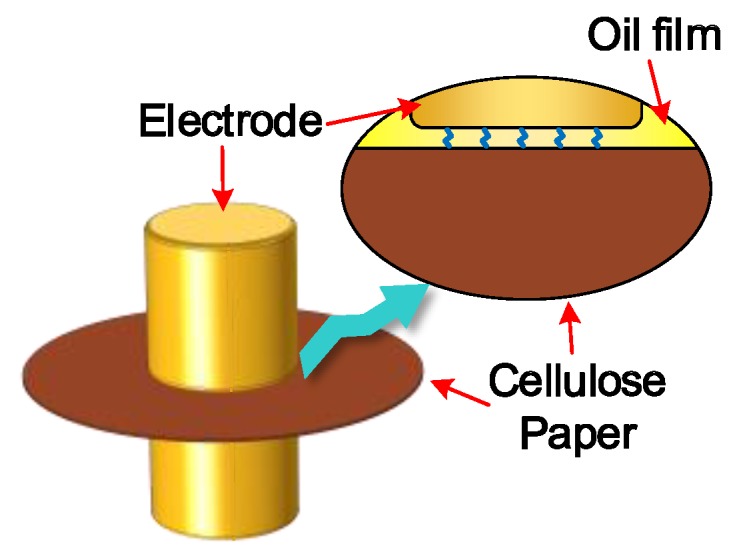
Oil film between electrode and cellulose paper.

The surface roughness of both OIP and NOIP subjected to repeated impulses was much higher than that of the fresh OIP sample. This indicates that repeated impulses can cause damage to the surface of dielectric. According to our previous study, since the relative permittivity of oil is lower than that of cellulose paper, the electric field in the oil film between the electrode and cellulose paper (Figure 19) is extremely high when impulse voltage is applied to the sample [52]. Therefore, partial discharge occurs in this oil film, which causes damage to the surface and variation in the surface morphology [22,26].

Please note that the surface roughness increases with an increasing nanoparticle concentration, which indicates that nanoparticles contribute to the damage of the OIP surface. This is because the relative permittivity of NOIP increases with the nanoparticle concentration, as demonstrated previously [53]. As such, the relative permittivity difference between oil film and cellulose paper becomes larger when the nanoparticle concentration increases. The electric field in the oil film thereby rises and partial discharge becomes more severe, which leads to the increase in the surface roughness as nanoparticle concentration increases.

#### 4.3.2. Micropore Structure

By measuring the pressure and volume of the mercury, we can calculate the diameter distribution, pore surface area, and porosity of OIP/NOIP. Assuming the radius of the micro pores in OIP and NOIP is *r*, contact angle is *θ*, pressure is *p*, surface tension of mercury is *γ*, the length of the micro pores is *L*, volume change of mercury is Δ*V*, and the surface area of the micro pores is *S*, we have the following equation:

(7) $$p\pi r^{2}L=\frac{2\gamma \pi rL}{\cos\theta }=p\Delta V$$

Then, we can obtain:

(8) $$r=\frac{2\gamma /\cos\theta }{p}$$

The surface area of the micro pores is expressed as:

(9) $$S=\frac{p\Delta V}{\gamma /\cos\theta }$$

**Figure 20:**
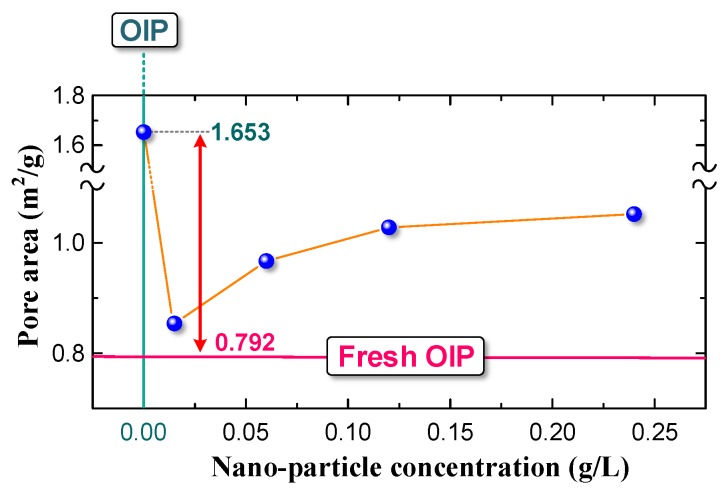
Pore area in fresh OIP and OIP/NOIP subjected to 500 impulse voltage.

We determined the pore area in fresh OIP and OIP and NOIP subjected to 500 impulse voltage with an amplitude of impulse voltage of 15 kV, as shown in Figure 20.

The pore area of OIP subjected to repeated impulses (1.653 m^2^/g), directly related to the number of pores, was much higher than that of fresh OIP (0.792 m^2^/g). This phenomenon indicates that repeated impulses can damage the dielectric and change the microstructure of OIP. However, when we impregnated the cellulose paper with nano-modified oil, the nanoparticles had a significant positive effect on the dielectric by reducing the accumulative damage. The pore area of NOIP subjected to repeated impulses was obviously lower than that of OIP subjected to repeated impulses. This may be because the nanoparticles in the NOIP can capture free electrons when repeated impulse voltage is applied. This contributes to weakening the accumulative damage caused by the collision of charged particles and electrons. In addition, nanoparticles can improve the relative permittivity of oil in the pores contained in the NOIP, thereby reducing the electric field in oil pores. This also helps to suppress partial discharge in the dielectric and reduce the accumulative damage caused by repeated impulses. Given the above, we concluded that nanoparticles can reduce the accumulative damage caused by repeated impulses and improve the OIP’s resistant ability against repeated impulses.

**Figure 21:**
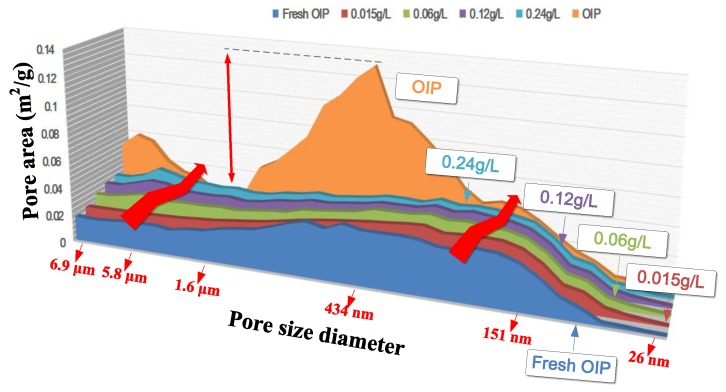
Diameter distribution of micro pores in OIP and NOIP.

However, the pore area of NOIP increased as the nanoparticle concentration increased. To explain this phenomenon, we determined the diameter distribution of micropores in fresh OIP and OIP/NOIP subjected to 500 impulse voltage, as shown in Figure 21.

Please note that as the nanoparticle concentration increased, the number of pores in NOIP, especially for the papers whose diameter was several μm or hundreds of nm, increased gradually. However, the number of pores whose diameters were less than 80 nm barely changed. This is because nanoparticles have a large specific surface area. The existence of nanoparticles in the dielectric can increase the surface area of pores. Some of the nanoparticles adsorb on the wall of micropores that cannot be displaced out of the sample in the acetone extracting treatment. All the samples in this test were immersed in acetone for 72 h and then dried at 90 °C for another 72 h before the micropore structure measurement. Therefore, the higher the nanoparticle concentration, the more nanoparticles stay in the micropores, and thus the larger the pore area. Since the nanoparticles can hardly enter nanopores whose diameter is too small, the number of pores whose diameter is less than 80 nm barely changes with an increasing nanoparticle concentration (Figure 21).

**Figure 22:**
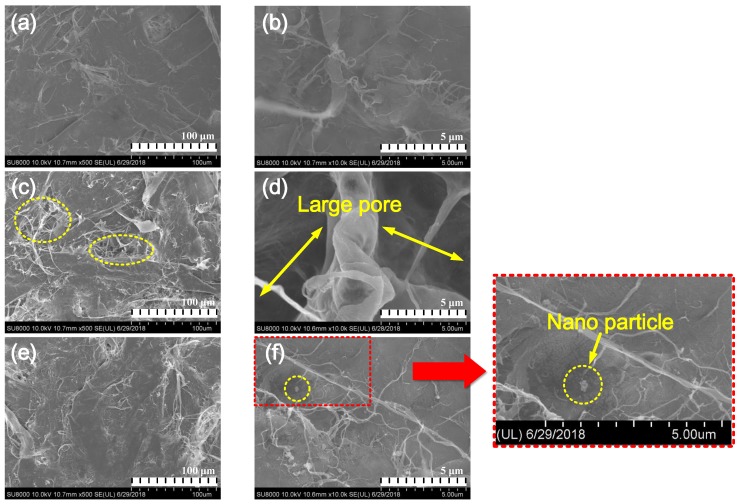
Scanning electron microscope (SEM) images of OIP and NOIP: (**a**,**b**) fresh OIP (magnified 500 and 10,000 times, respectively), (**c**,**d**) fresh OIP subjected to 500 impulses (magnified 500 and 10,000 times, respectively), (**e**,**f**) NOIP subjected to 500 impulses (magnified 500 and 10,000 times, respectively).

To further study the micropore structure of NOIP, we observed the inside morphology of both OIP and NOIP subjected to 500 impulse voltage. The surface layers of the samples were removed to facilitate inside observation. All the test samples were immersed in acetone after accumulation test. The test results are shown in Figure 22.

Please note that the structure of fresh OIP is very compact. The gap between cellulose fibers are small, as shown in Figure 22a,b. However, when apply repeated impulses to the dielectric, large gaps emerged between the fibers. Several pores were detected as shown in Figure 22c. The diameter of large pores reached several micrometers (Figure 22d). This indicates that repeated applications of impulse voltage affect the microstructure of OIP and lead to an increase in the micropores. In Figure 22e, some small gaps between the fibers in the NOIP area visible, but the amount and size of the micropores is obviously smaller than that of OIP subjected repeated impulses. As shown in Figure 22f, we detected some nanoparticles on the cellulose fibers. They are considered to remain in the cellulose paper after acetone extracting treatment, which further proves our previous analysis in Section 4.3.2.

## 5. Conclusions

The positive U_50_ of NMTO increased first and then decreased slightly with increasing nanoparticle concentration. The negative U_50_ of NMTO decreased with the increasing nanoparticle concentration. Nano-TiO_2_ particles had a limited effect on the U_50_ of NOIP. However, nano-TiO_2_ particles can dramatically improve the resistant ability of NOIP against repeated impulses. When the amplitude of the applied impulse voltage was 15 kV, the *N* was 62.5% higher for NOIP (0.25 g/L nanoparticle concentration) compared with that of OIP.

Nano-TiO_2_ particles can suppress the accumulation of space charge in the oil paper insulation, which weakens the electric field distortion in the dielectric, which improves the resistant ability against repeated impulses. We observed the following important phenomena. The space charge density of NOIP was much lower than that of OIP. Deep traps were introduced in the NOIP as the nanoparticle concentration increased; the deep trap density increased initially and then decreased slightly, whereas the shallow trap density decreased slightly. Deep traps in NOIP both suppress the charge injection and suppress charge accumulation by scattering effect. Negative charges were detected on the paper interface between the second and third layers in the NOIP sample, which is contrary to the phenomenon in OIP. The space charge decay rates of the NOIP samples were higher than in the OIP sample.

Repeated impulses can cause accumulative damage to the cellulose paper surface, thereby leading to the increase in the surface roughness. Due to the increase in the relative permittivity of NOIP caused by nanoparticles, the electric field in the oil film between the electrode and cellulose paper decreased. This results in the surface roughness of NOIP increasing with increasing nanoparticle concentration. Repeated impulses can damage the cellulose paper and change the microstructure of the cellulose paper, which is directly shown by the increase in the amount of micropores in the cellulose paper. Nanoparticles can reduce the accumulative damage caused by repeated impulses and decrease the increase in the number of micropores in the cellulose paper.

## 6. Follow-Up Work and Research Progress

In this paper, we determined the failure characteristics of both OIP and NOIP and revealed the effects of nano-TiO_2_ particles on the space charge behavior and accumulative damage characteristics. The NOIP only shows a good resistant capability against repeated impulse voltages, but its breakdown voltage barely changes. In the near future, we intend to focus on the overall improvement of nano-modified paper, which important for improving the oil paper insulation system properties.

In addition, some of the latest research advances are essential to share with readers:

To find a NOIP with a good ability to resist repeated impulses, we successively tried Al_2_O_3_, Fe_3_O_4_, ZnO, SiO_2_, and C_60_. The breakdown voltage of NOIP modified by the above nanoparticles barely changed. The capability against repeated impulses was better than that of fresh OIP, to some extent. However, the improvement was still relatively limited compared with NOIP modified by nano-TiO_2_. 

If we dramatically increased the nanoparticle concentration, although the stability could not be guaranteed at all, the breakdown voltage of NOIP with extremely high nanoparticle concentration was even higher than that of fresh OIP. This is a preliminary test result. More tests and analysis are needed to explore the stability of new-found NOIP and to reveal its modification mechanisms.

By adding some nanoparticles into paper pulp, we directly manufactured nano-modified cellulose paper. The electrical performance of nano-modified paper has been preliminarily proved to be better than fresh paper.

## Figures and Tables

**Table 1:** Parameters for the electrodes.

Electrode	Diameter	Height	Edge Radius	Tip Radius	Material	Gap Distance
Needle	-	-	-	60 ± 10 μs	Brass	4 mm
Plate	60 mm	8 mm	2.5 mm	-	Brass	
Column	25 mm	25 mm	1 mm	-	Brass	-
